# Multipronged activity of combinatorial miR-143 and miR-506 inhibits Lung Cancer cell cycle progression and angiogenesis ***in vitro***

**DOI:** 10.1038/s41598-018-28872-2

**Published:** 2018-07-12

**Authors:** A. K. M. Nawshad Hossian, Md. Sanaullah Sajib, Paul E. Tullar, Constantinos M. Mikelis, George Mattheolabakis

**Affiliations:** 1 Department of Basic Pharmaceutical Sciences, School of Pharmacy, University of Louisiana at Monroe, Monroe, Louisiana USA; 2 Department of Biomedical Sciences, School of Pharmacy, Texas Tech University Health Sciences Center, Lubbock, Texas USA; 3 Department of Obstetrics and Gynecology, School of Medicine, Texas Tech University Health Sciences Center, Lubbock, Texas USA

## Abstract

Lung cancer (LC) is the leading cause of cancer-related deaths. Downregulation of CDK1, 4 and 6, key regulators of cell cycle progression, correlates with decreased LC cell proliferation. Enforced expression of miRNAs (miRs) is a promising approach to regulate genes. Here, we study the combinatorial treatment of miR-143 and miR-506 to target the CDK1, 4/6 genes, respectively. We analyzed the differential expression of CDK genes by qPCR, and western blot, and evaluated changes in the cell cycle distribution upon combinatorial treatment. We used an antibody microarray analysis to evaluate protein expression, focusing on the cell cycle pathway, and performed RNA-sequencing for pathway analysis. The combinatorial miR treatment significantly downregulated CDK1, 4 and 6 expression, and induced a shift of the cell cycle populations, indicating a G1 and G2 cell cycle block. The two miRs induces strong cytotoxic activity, with potential synergism, and a significant Caspase 3/7 activation. We identified a strong inhibition of tube formation in the presence or absence VEGF in an *in vitro* angiogenesis model. Together with the pathways analysis of the RNA-sequencing data, our findings establish the combinatorial miR transfection as a viable strategy for lung cancer treatment that merits further investigation.

## Introduction

miRNAs (miRs) are small non-coding RNAs consisting of 19–25 nucleotides^1^. These unique molecules regulate at least 30% of all human gene expressions, either by translational repression or target messenger RNA destabilization. For gene regulation to take place, miRs require base-pair complementarity between the targeted messenger RNA (mRNA) and the seed region of the miR, with their activity relying on the cell’s natural RNA interference mechanism^2,3^.

Researchers have identified more than 5,000 miRs, from which >3,700 have been added to our knowledge within the last couple of years alone^4^. The clinical significance of miRs can be appreciated by their versatility to regulate multiple pathways, since each miR sequence is able to bind to/target multiple mRNAs^4–7^. Not surprisingly, miRs regulate tumor formation, growth and metastasis, and are classified as either oncogenes or tumor suppressors^8^. Thus, miRs have become an important tool or/and target for cancer therapy.

Lung cancer is a devastating disease, with more than 1.6 million of lung cancer-related deaths recorded per year world-wide^9^, and approximately 85% of the cases attributed to non-small cell lung cancer (NSCLC)^10^. Despite the recent advents of therapeutic options, the 5-year survival rate remains low (~15%)^11,12^. Lung cancer cells are characterized by rapid and unregulated proliferation. At the core of the four sequential stages (G1, S, G2, M) of the cell cycle progression is the differential expression and activation of cyclin-dependent kinases (CDKs) that permit or drive the cell cycle progression^13,14^. Among the different CDKs, CDK1, CDK2, CDK4 and CDK6 are primarily associated with the cell cycle progression^15^. Briefly, the S and M phases potentiate the successful cell division^16^, with the activated CDK1 exerting its activity during the G2/M transition, and CDK4/6 exerting their activity during the G1/S transition^13,17^.

**Figure 1:**
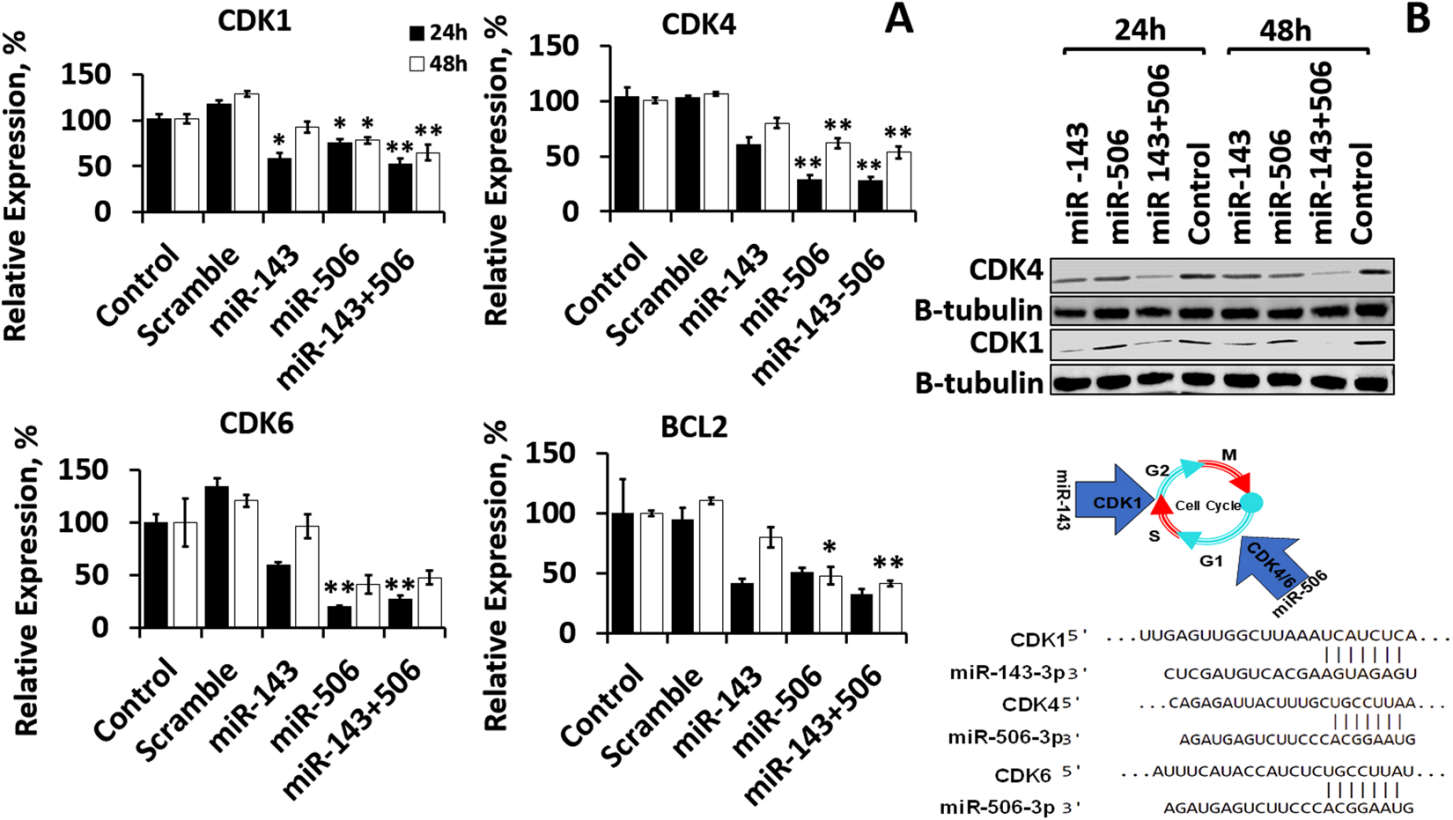
miR-143 and/or miR-506 transfection induced significant downregulation of CDK1, CDK4, CDK6 and BCL2 expression in A549 lung cancer cells, at 24 and 48 h post transfection. (**A**) mRNA relative expression for CDK1, CDK4, CDK6 and BCL-2, as detected by qPCR. All expressions were normalized to control (untreated) cells. GAPDH was used as reference gene. (**B**) Western Blot analysis of protein expression for CDK1 and CDK4. *p < 0.05; **p < 0.01 vs. control. Transfection took place as described in the *Methods* section.

Existing literature indicates that miR-143 and miR-506 are downregulated in NSCLC cells and can individually affect cell proliferation^3,18^. Utilizing predicting software for identifying potential miR targets (www.targetscan.org)^19^, we determined that miR-143 and miR-506 have base pair complementarity with the CDK1 and CDK4/6 mRNAs, respectively (Fig. 1), demonstrating a potential to combinatorially regulate the cell cycle on different stages. In this study, we report that the combinatorial treatment of A549 cells with the two miRs induces strong downregulation of CDK1, 4 and 6, and causes strong cell cycle arrest, accompanied with apoptotic and cytotoxic activity, and caspase 3/7 activation. Microarray and RNA-sequencing pathway analyses indicate that a cascade of gene alterations takes place, correlating with a strong cell cycle arrest. Furthermore, we determined that the combinatorial treatment significantly inhibited tube formation in an *in vitro* angiogenesis model, endowing the proposed treatment with multifaceted activity against the tumor cells and the tumor microenvironment.

## Results

### Combinatorial treatment of miR-143 and -506 significantly downregulates CDK1, CDK4 and CDK6 gene expression

We determined the CDK1, CDK4 and CDK6 mRNA expression alterations due to transfection with miR-143 and/or miR-506 in A549 lung cancer cells using quantitative real-time PCR (qRT-PCR). The combinatorial therapy of miR-143 and miR-506 significantly downregulated CDK1, CDK4 and CDK6 expression by 47% (p < 0.01), 71% (p < 0.01) and 73% (p < 0.01), at the 24 h time point, and by 35% (p < 0.01), 46% (p < 0.01) and 53%, respectively, at the 48 h time point (Fig. 1A). miR-143 alone downregulated CDK1 by 42% (p < 0.05), CDK4 by 40% (no p value) and CDK6 by 41% (no p value) at the 24 h time point, and all expression levels normalized for the three genes at the 48 h time point. miR-506 alone downregulated the expression of CDK1 by 24% (p < 0.05), CDK4 by 71% (p < 0.01), and CDK6 by 80% (p < 0.01), at the 24 h time point, while downregulated CDK1 by 21% (p < 0.05), CDK4 by 38% (p < 0.05), and CDK6 by 60% (no p value), at the 48 h time point (Fig. 1A).

We identified that the miR-143 and miR-506 co-treatment significantly downregulated the anti-apoptotic BCL2 expression by 68% (no p value) and 59% (p < 0.01), at the 24 and 48 h time points, respectively (Fig. 1A). The individual miRs did not demonstrate similar behavior, with only miR-506 at the 48 h time point inducing a 53% (p < 0.05) downregulation of BCL2 expression. All data were normalized to untreated controls.

We further evaluated at the post-translational level (protein) the downregulation of CDK1 and CDK4 in A549 cells transfected with miR-143 and/or miR-506. Western Blot analysis confirmed a strong downregulation for the CDK1 and CDK4 protein levels at the 24 and 48 h time points post-transfection due to the combinatorial miR treatment, with CDK4 expression being almost completely suppressed 48 h post transfection. CDK1 protein levels demonstrated a more modest downregulation. The individual miRs did not demonstrate similar downregulation, although the miR-506 downregulated CDK4 at the 48 h time point and miR-143 downregulated CDK1. We used β-Tubulin as the reference protein (Fig. 1B). There is a time difference of the observed maximum gene downregulation, as detected by the qPCR at the mRNA level and by the Western Blot analysis at the protein level, with the maximum effect being observed at the 24 h time point for the former, and at the 48 h time point for the latter.

### miR-143 and -506 combinatorial treatment halts cell cycle progression

**Figure 2:**
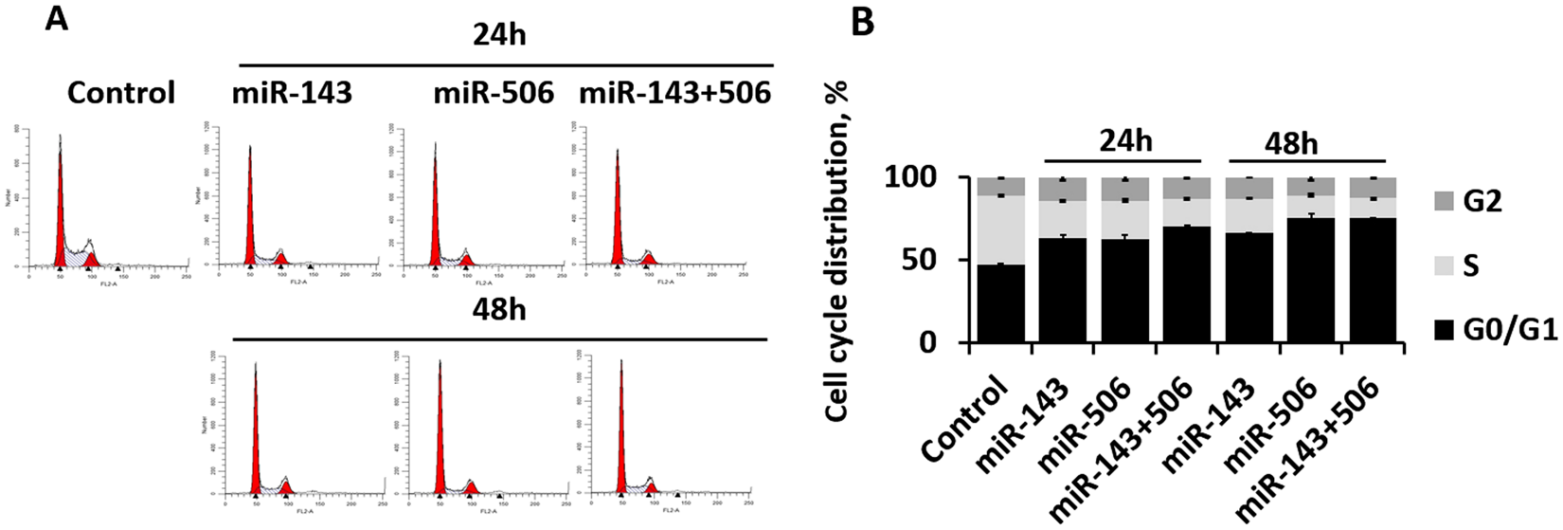
miR-143 and miR-506 co-treatment induces strong cell cycle arrest, and increases the cell populations at the G0/G1 and G2 phases. (**A**) Representative cell distribution flow cytometry charts of A549 cells treated with or without miR-143 and/or miR-506, 24 and 48 h post transfection. (**B**) Percentages of the cells in each phase of the cell cycle, as determined by the flow cytometric analysis.

We demonstrated that transfection of A549 cells with the combinatorial miR treatment significantly downregulated the gene expression of CDK1, CDK4 and CDK6, which are critical for the successful transition through the different cell cycle stages. Subsequently, we evaluated changes in the cell distribution according to the different cell cycle phases, due to the miR transfection. Utilizing a standard cell cycle analysis with propidium iodide staining and flow cytometry^20^, the combinatorial treatment of miR-143 and -506 induced significant (p < 0.05) changes on the cell-cycle cell distribution. At 24 h post transfection, the cell population in the G0/G1 phase of the cell cycle increased to 71% (p < 0.05, all p values compared to untreated cells) due to the combinatorial miR treatment, compared to 47% for untreated, 63% (p < 0.05) for miR-143 alone, and 63% (p < 0.05) for miR-506 alone, while at 48 h post transfection, the cell population in the G0/G1 phase of the cell cycle increased to 75% (p < 0.05) due to the combinatorial miR treatment, compared to 66% (p < 0.05) for miR-143, and 75% (p < 0.05) for miR-506 alone (Fig. 2, Supplementary Table 1). At 24 h post-transfection, the S phase cell population dropped to 16% (p < 0.05) due to the combinatorial miR treatment, compared to 41% for untreated cells, 22% (p < 0.05) for miR-143 alone, and 23% (p < 0.05) for miR-506 alone, while at 48 h post transfection, the combinatorial treatment reduced the S phase cell population to 12% (p < 0.05), compared to 21% (p < 0.05) for miR-143 alone, and 13% (p < 0.05) for miR-506 alone. Transfection with miR-143 or miR-506 alone increased the cell population in the G2 phase, to 14% and 14% at the 24 h, and 13% and 11% at the 48 h, respectively, compared to 11% for untreated cells (no p value was achieved). The combinatorial treatment increased the cell population in the G2 phase to 13% for the 24 h and 13% for the 48 h time points (p < 0.05 for both, Supplementary Table 1).

### miR-143 and miR-506 combinatorial treatment induces strong apoptotic and cytotoxic effect

**Figure 3:**
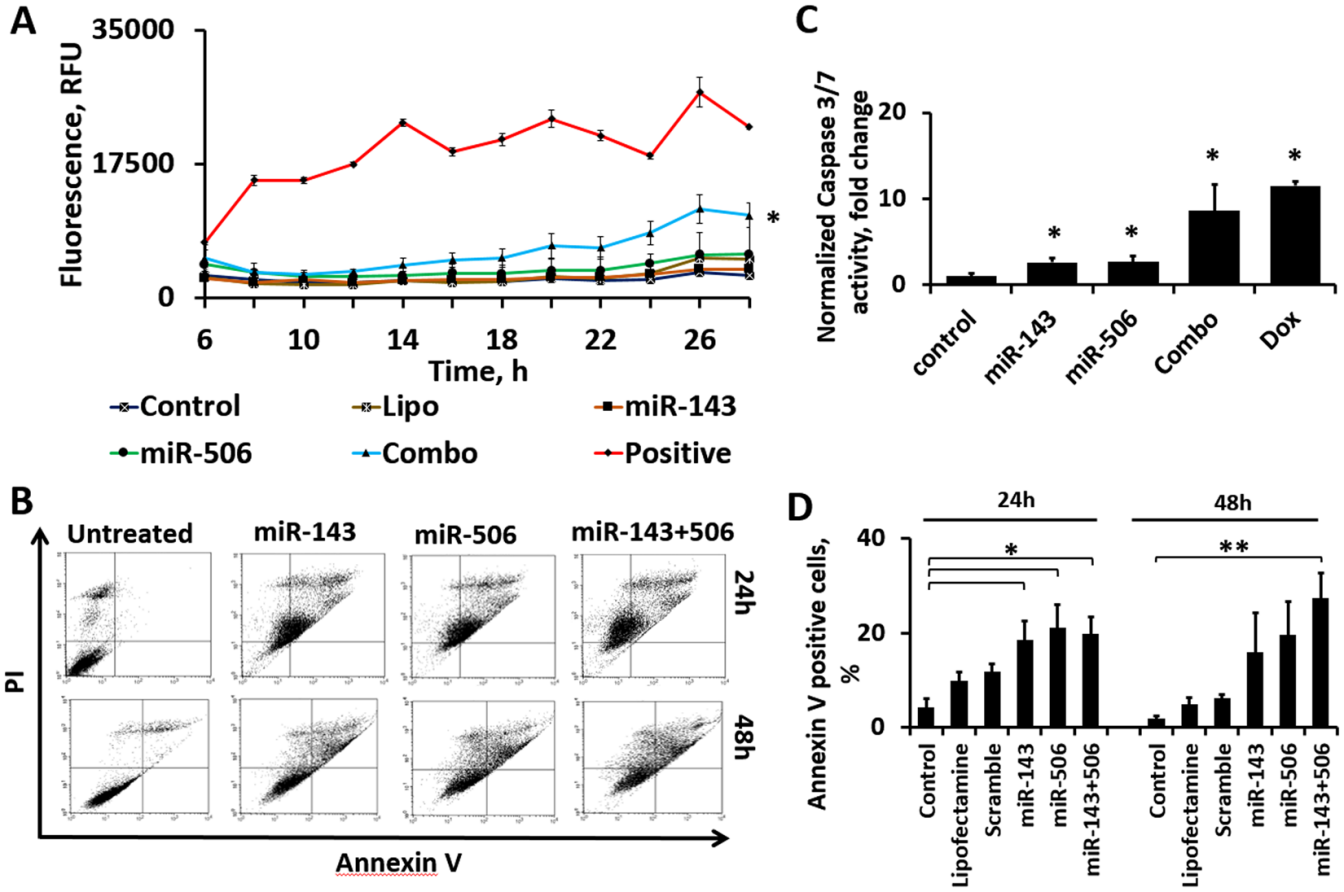
The combinatorial miR treatment induced strong cytotoxic and apoptotic effect, along with Caspase activation (**A**) Potential synergism is illustrated by the combination’s (Combo) cytotoxicity being larger than the addition of the cytotoxicity of the individual miRs alone. Fluorescence intensity, which correlates to number of dead cells, was measured till 33 h post transfection. Lipofectamine with Scramble (Lipo), and untreated cells (Control) were used as negative controls. Positive control (Positive) was provided by the vendor. *p < 0.05 of combo vs. Control, miR-143, miR-506. (**B**) FACS Annexin V/PI analysis of cell apoptosis treated with individual mimics, and combination at 24 and 48 h compared to untreated cells. (**C**) Caspase-3/7 activity of A549 cells treated with individual and combination of miR, at 24 h. Cells without any treatment (Negative control-NC), with Lipofectamine, and with no-target control miRNA (scramble) were used as experimental controls. Doxorubicin (DOX, equimolar) was used as positive control. **p < 0.01 relative to NC. (**D**) Annexin V positive A549 cells, following transfection with miR-143 and/or miR-506 at 24 and 48 h post transfection.

We utilized the Celltox green assay kit to evaluate the cytotoxic activity of miR-143 and miR-506 against A549 cells *in vitro*. The method correlates fluorescence intensity to the number of dead cells. The combinatorial miR treatment demonstrated a strong cytotoxic activity, with the effect becoming more prominent at ~12 h post-transfection and steadily increasing beyond that (Fig. 3A). More importantly, the combinatorial treatment exhibited significantly stronger cytotoxic activity compared to either miR-143 or miR-506 alone, with the combinatorial treatment having >3-fold higher cytotoxicity vs. miR-143 and >2-fold higher vs. miR-506 (p < 0.05 for combination vs. miR-143, miR-506 – one tailed; and control – two tailed).

We evaluated the capacity of the combinatorial treatment to induce apoptotic activity against A549 lung cancer cells, using a Annexin V/PI Early Apoptosis Detection Kit (Cell Signaling), following an established protocol^20^. The combinatorial miR treatment induced strong apoptotic activity and necrotic behavior *in vitro* at the 48 h time point. More specifically, at the 24 h time point post transfection, 45% (p < 0.05 vs. control; no p-value vs. scramble) of the cells demonstrated late apoptotic or necrotic behavior for the combinatorial treatment, compared to 32% (p < 0.05 vs. control; no p-value vs. scramble) for miR-143 and 37% (p < 0.05 vs. control; no p-value vs. scramble) for miR-506 alone, while at the 48 h time point, 57% (p < 0.001 vs. control, scramble; p < 0.05 vs. miR-143, -506 alone) of the cell population exhibited late apoptotic or necrotic behavior for the dual miR treatment, compared to 31% for miR-143 alone (p < 0.01 vs. control, scramble) and 46% (p < 0.01 vs. control, scramble) for miR-506 alone (Fig. 3B, Table 1). The apoptotic activity of the two miRs can also be appreciated by the significantly increased number of Annexin V positive cells, a marker associated with apoptosis, with the combinatorial treatment sustaining prolonged and significantly higher number of Annexin V positive cells at the 48 h, compared to the individual miR treatments. Lipofectamine alone or scramble with lipofectamine were used as negative controls (Fig. 3).

**Table 1:** Annexin V/PI apoptosis analysis of A549 cell populations, as determined by flow-cytometric quantification (Average of triplicates ± SEM).

	No apoptosis	Early apoptosis	Late apoptosis and necrosis
24 h			
Control	87.9 ± 3	1.3 ± 0.6	10.8 ± 2.4
Lipofectamine	73.9 ± 9	1.8 ± 0.5	24.3 ± 8.5
Scramble	67.9 ± 9.5	2 ± 0.9	30.1 ± 8.6
miR-143	62.3 ± 2.7	5.5 ± 1.8	32.1 ± 4.4
miR-506	55.7 ± 4.6	7.2 ± 2.7	37.2 ± 7.3
miR-143/506	50.6 ± 8.3	4.3 ± 0.7	45.2 ± 8.4
48 h			
Control	91.8 ± 0.7	0.3 ± 0	7.9 ± 0.7
Lipofectamine	84.6 ± 2.5	0.6 ± 0.3	14.8 ± 2.2
Scramble	85 ± 2.4	0.7 ± 0.4	14.3 ± 2.2
miR-143	67.4 ± 4.4	1.2 ± 0.1	31.4 ± 4.3
*miR-506*	52.5 ± 1.4	1.3 ± 0.1	46.2 ± 1.3
*miR-143/506*	41.6 ± 2.8	1.3 ± 0.1	57.1 ± 2.7

### Combinatorial therapy induces Caspase 3/7 activation

We transfected A549 cells with miR-143 and/or miR-506, and analyzed the Caspase-3/7 activity. We detected a >8-fold increase (p < 0.05) of caspase levels at the 24 h time point post-transfection, compared to the untreated cells (control), due to the transfection with the combinatorial miR treatment, while the individual miRs alone demonstrated a more modest caspase activation, with miR-143 or miR-506 alone inducing a 3-fold increase (p < 0.05) of caspase activation. Interestingly, equimolar concentration of doxorubicin (100 nM, positive control) induced a 11-fold increase in caspase levels (Fig. 3C).

### Alterations of cell cycle’s protein expressions due to combinatorial miR treatment indicate cell cycle arrest

**Figure 4:**
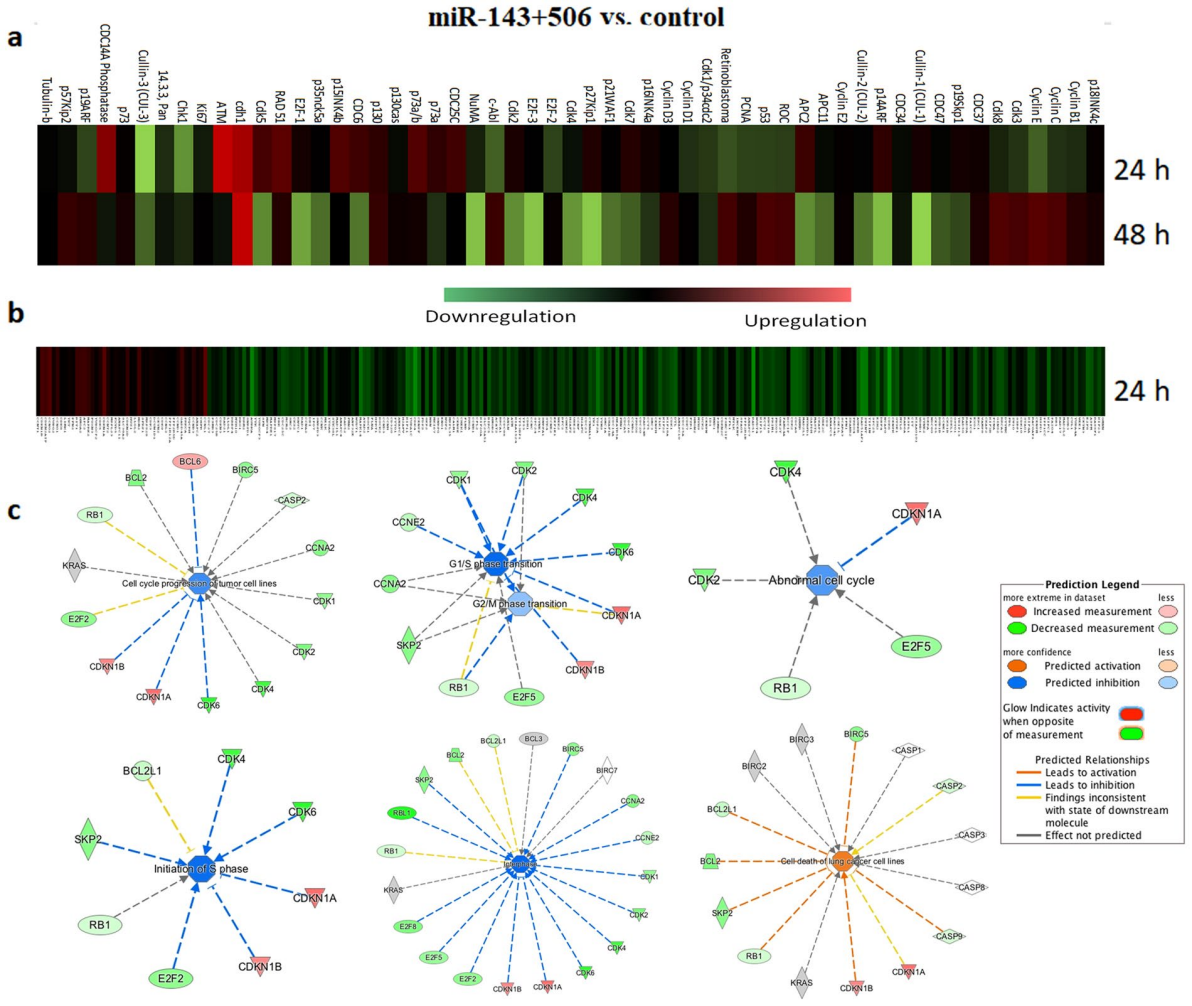
Microarray, and RNA-sequencing analysis of the cell cycle pathway indicated strong dysregulation for cells treated with the combinatorial miR-143 and 506 treatment, favoring cell cycle halt. (**a**) Heatmap of proteins associated with the cell cycle pathway were analyzed using a cell cycle specific antibody microarray. 60 proteins expression were evaluated 24 and 48 h post transfection with combinatorial therapy. (**b**) Heatmap of genes associated with the cell cycle pathway, as analyzed by RNA-sequencing, evaluated 24 h post transfection. (**c**) IPA of genes associated with cell cycle, from the RNA-sequencing data, confirm a strong cell cycle arrest in both G1/S and G2/M transitions due to treatment.

To identify the effects of miR-143 and miR-506 combinatorial treatment on the expression of relevant activators or inhibitors for the cell cycle pathway, we utilized a microarray system to study the protein expression of cell cycle regulating targets. Microarray analysis is a widely used technique to evaluate the expression of several proteins semi-quantitively. As shown in the heatmap of the relative protein levels for untreated vs. treated cells (Fig. 4a), extensive alteration of gene expression took place at the 24 and 48 h post transfection time points. Briefly, the combinatorial miR therapy downregulated CDK1 and CDK4 protein levels, as well as important proteins for cell division, such as the E2F family. On the other hand, the tumor suppressor protein p53 and retinoblastoma^21,22^ were upregulated 48 h post transfection. Tumor suppressors, such as p16^Ink4a^, p21^WAF1/CIP1^, sp27^KIP1^, and p15^Ink4b^, which act by negatively regulating CDK activity^23–26^, were upregulated 24 h post transfection. In Table 2, we outline proteins that demonstrated >10% of differential expression between treated and untreated samples, along with their biological activity. Off-note, we did not include in Table 2: (a) CDK3, whose activity on the cell cycle has not clearly been identified; (b) CDK8, a transcriptional regulator, whose activity has been designated as both tumor promoter and suppressor, and a p53 co-activator^27–30^; and (c) any of the cyclins.

**Table 2:** Genes and their activity on the cell cycle, which demonstrated >10% up-/down-regulation, as identified by the microarray analysis of A549 cells transfected with miR-143/506, for 24 and/or 48 h post transfection, compared to control.

Upregulated			Downregulated		
*Gene*	*Activity*	*Ref*.	*Gene*	*Activity*	*Ref*.
p57Kip2	Potent tight-binding inhibitor of several G1 cyclin complexes, and a negative regulator of cell proliferation	^60^	p19Skp1	Essential element of the cyclin A-CDK2 S phase kinase	^61^
p53	Tumor suppressor gene	^21^	Cullin-1, Cullin-3	Upregulation promotes cell proliferation	^62^
Retinoblastoma (Rb)	Active RB halts cell cycle progression – Tumor suppressor	^22^	APC11	Knockdown of APC11 contributes to the delay in cell cycle	^63^
c-Abl	Overexpression of c-Abl inhibits cell growth and leads to G1 arrest	^64^	CDK4	Targeted protein – Promotes cell cycle progression	^37^
E-Cadherin (cdh1)	Plays an important role in mitotic regulation.	^65^	E2F-1 E2F-2 E2F-3	E2F family members bind DNA and promote DNA replication	^66, 67^
p19ARF	Cell cycle inhibitor	^68^	CDK5	Contributes to tumor proliferation, migration, angiogenesis, and is linked to chemotherapy resistance	^69^
ATM	Induces cell cycle arrest and apoptosis	^70^	CDK2	Promotes cell cycle progression	^37^
			NuMA	Essential for the organization and stabilization of spindle poles from early mitosis	^71^
			CDC6	Essential regulator of DNA replication in eukaryotic cells	^72^
			p35nck5a	A neuron-specific activator of Cdk5	^73^

Prompted by these results, we expanded and evaluated in-depth possible pathway behaviours through RNA-sequencing. RNA-sequencing can accurately and reliably determine the expression of large number of genes post-trascriptionally.

Samples were treated with miR-143 and miR-506, as above, and RNA was extracted and analyzed, 24 h post transfection. Relative expression between untreated (control) and combinatorially-miR-treated samples of A549 cells was determined. We developed heatmaps of the genes associated with the cell-cycle pathway (Fig. 4b), and other important pathways (Supplementary Fig. 5). Overall, the majority of the genes associated with the cell cycle pathway were downregulated, while a relatively more balanced differential expression (upregulated vs. downregulated) was observed for the other studied pathways. Indicatively, through RNA-sequencing, we identified the CDK1, 4 and 6 genes being downregulated by 48% (p-value < 0.001, FDR < 0.001), 68% (p-value < 0.001, FDR < 0.001), and 71% ((p-value < 0.001, FDR < 0.001), respectively, and BCL-2 downregulated by 54% (p-value < 0.001, FDR < 0.001). These results comfirm the data obtained by the qPCR analysis presented above.

**Figure 5:**
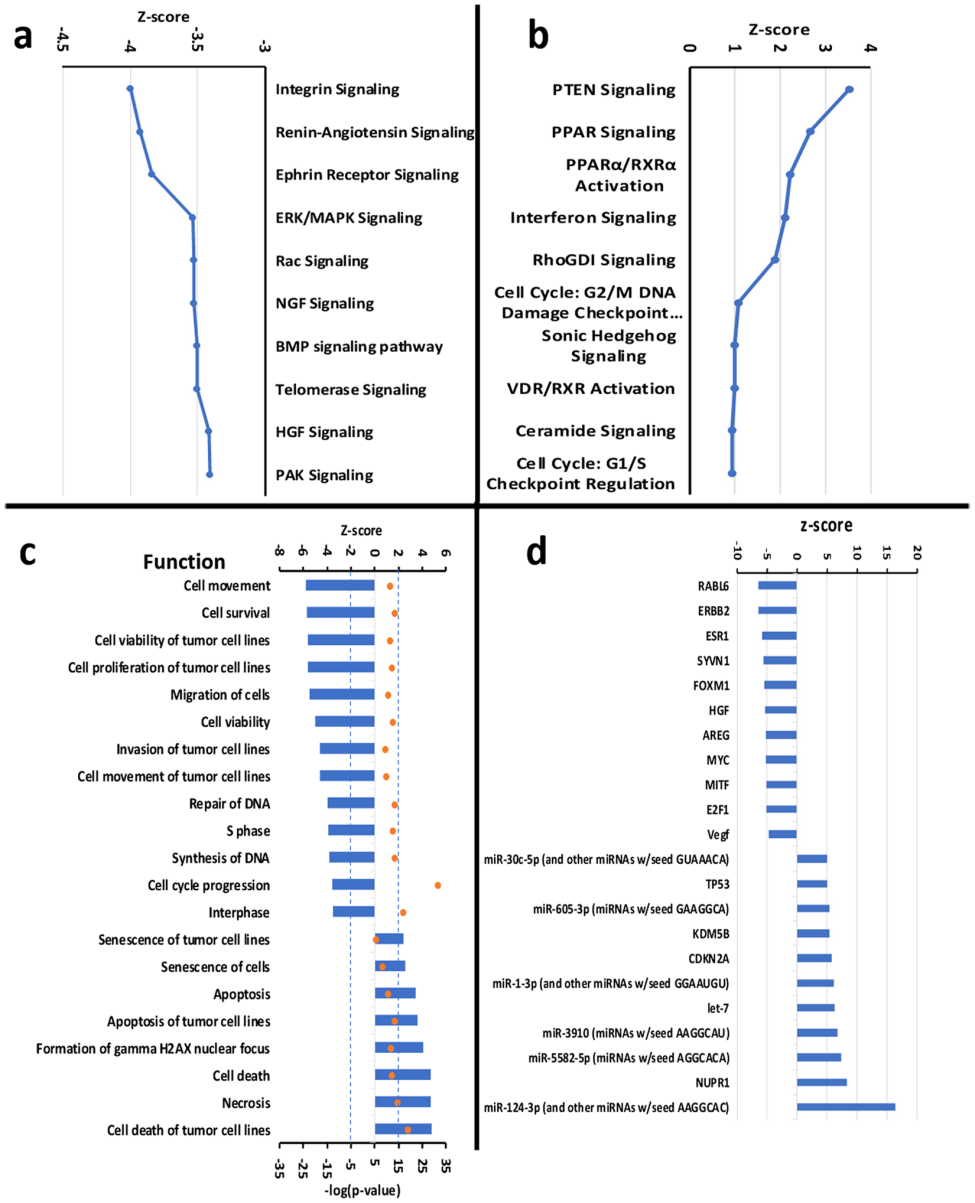
Analysis of canonical pathways, functions and upstream targets, along with predicted activation scores, due to the combinatorial treatment of miR-143 and miR-506, 24 h post transfection, as determined by RNA-sequencing and the IPA software. (**a**) 10 canonical pathways with the lowest z-score. (**b**) 10 canonical pathways with the highest z-score. (**c**) Predicted functions with the lowest and highest z-scores. (**d**) Predicted upstream regulators with the highest and lowest z-score (for all genes: −log(p-value) >1.5). All analyses took place on A549 cells transfected with the combinatorial miR, as described in the *Methods* section.

Due to the large number of the analyzed genes, we utilized the Ingenuity Pathway Analysis (IPA) software to evaluate pathway activity and to gain further insight into the biological mechanistic outcomes. IPA confirmed our earlier assessment and predicted a cell cycle arrest taking place in both G1/S and G2/M transitions, along with an overall inhibition of the cell cycle progression (Fig. 4c). Expanding outside the cell-cycle pathway, IPA identified additional pathways that were significantly affected from the combinatorial miR-143 and miR-506 treatment. In Fig. 5a,b, we present 10 pathways with the highest and 10 pathways with the lowest z-values, as were predicted by IPA. The activation score z indicates a pathway’s overall increase (positive values) or decrease (negative values) of mRNA levels and its primary purpose is to infer the activation status of implicated biological functions. p-Value (or −log(p-value)) is calculated using a right-tailed Fisher’s exact test (significance: p < 0.05 or −log(p-value) >1.3). In these data, we excluded pathways that were deemed unrelated to the disease. Overall, the IPA analysis indicated pathways and molecular mechanistic responses (either with positive or negative activation scores z) that associate with a strong apoptotic behaviour, and inhibition of the cell cycle progression. We analyze in more detail these pathways in the discussion section below.

A summarized analysis is provided by the software by predicting specific biological functions that should be observed based on the detected gene dysregulations due to the treatment, with the software assigning a z-score to such functions. According to the vendor’s instructions, z-score reflects an overall predicted activation/inhibition state of the biological function (negative values: inhibited; positive values: activated), and |z| >2 are considered significant^31^. IPA assigned the strongest negative scores to functions associated with cell survival, cell proliferation and cell movement (Fig. 5c). The functions with the highest z-score values include senescence of the cells, apoptosis, cell death and necrosis. These results confirm our earlier assessment of the treatment. Finally, IPA also provided with a list of predicted upstream targets, as presented in Fig. 5d.

### Combinatorial miR treatment inhibits endothelial tube formation *in vitro*

**Figure 6:**
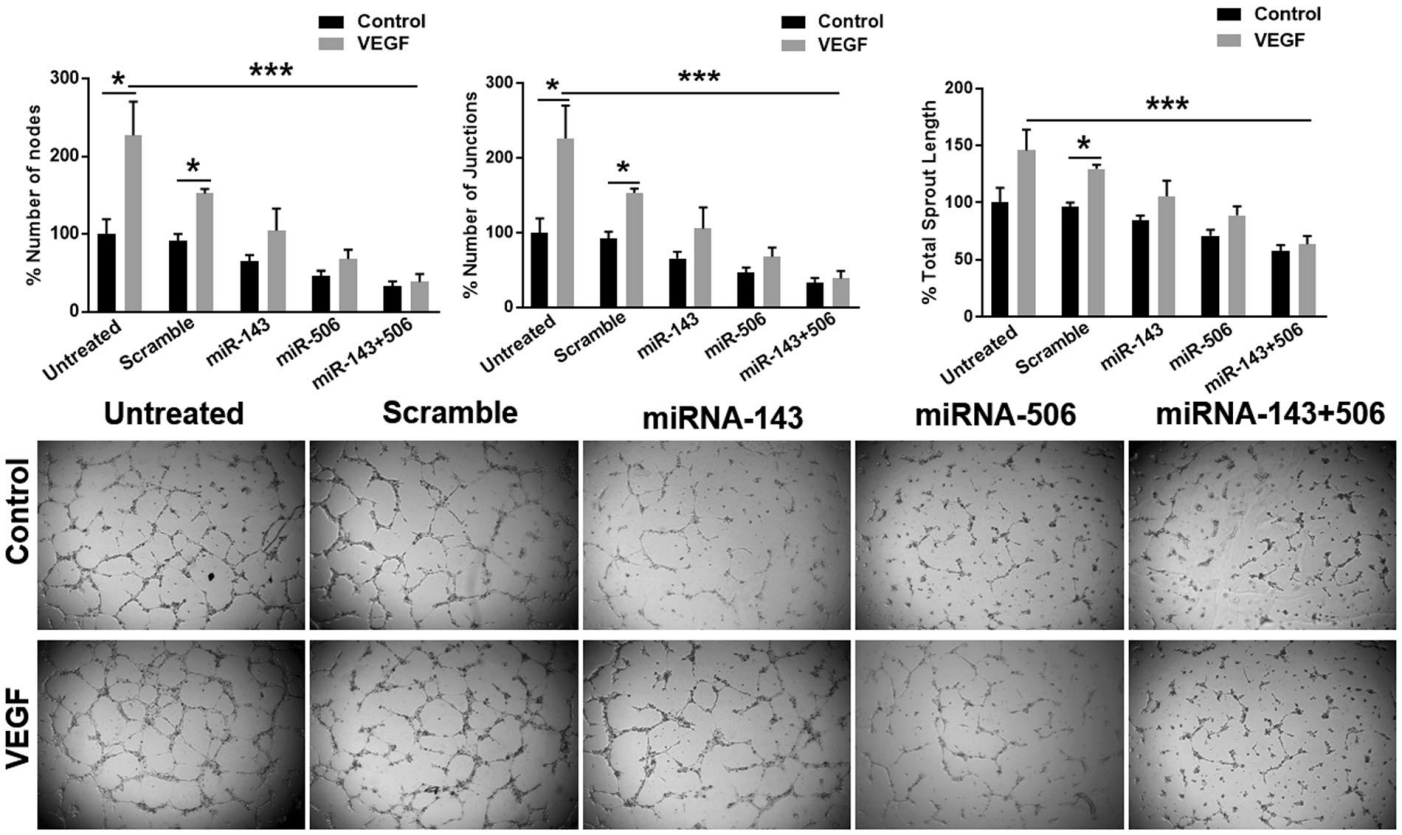
Combinatorial miR treatment inhibited tube formation *in vitro*. The tube formation assay was utilized as an *in vitro* angiogenesis model to assess the anti-angiogenic activity of the combinatorial miR-143 and miR-506 treatment on utilizing HUVECs, both on the basal, as well as on VEGF-induced angiogenesis.

The microarray analysis indicated a downregulation of the CDK5 levels upon miR-143/506 co-treatment (Fig. 4 and Table 2). CDK5 regulates endothelial migration and angiogenesis, and is recognized as a potential target for anti-angiogenic therapy^32^. Furthermore, the IPA software presented the biological functions of cell movement, cell migration, and invasion of tumor cells strongly negatively regulated with z-scores <−2. Finally, the IPA software indicated VEGF as a predicted upstream regulator, with z-score <−2 (Fig. 5d). Vascular Endothelial Growth Factor (VEGF) is an well-known angiogenic growth factor and is commonly the main target of anti-angiogenic therapies^33,34^. These results prompted us to evaluate the anti-angiogenic effect of the treatment, using an *in vitro* angiogenesis model. The combination treatment of miR-143 and miR-506 blocked endogenous angiogenesis, as assessed from the decrease of the number of nodes by 67% (p < 0.05, all p values are compared to untreated cells unless stated otherwise), the number of junctions by 67% (p < 0.05) and the overall sprout length by 42% (p < 0.05), in the *in vitro* tube formation assay, using Human Umbilical Vein Endothelial Cells (HUVECs, Fig. 6)^35^. Individually, the miR-506 alone was slightly less efficient compared to the combinatorial miR treatment (no p value miR-506 alone vs. combo), reducing the number of nodes by 54% (p < 0.05), the number of junctions by 54% (p < 0.05), and the overall sprout length by 29% (p < 0.05), whereas the miR-143 demonstrated a significantly less potent effect in inhibiting tube formation compared to the combination therapy (p < 0.05 of miR-143 alone vs. combo), by reducing the number of nodes by 36%, the number of junctions by 34%, and the overall sprout length by 16% (no p significance vs. control). Overall, the combinatorial treatment demonstrated the strongest effect in all cases.

We identified that the combinatorial treatment of miR-143 and miR-506, along with the basal angiogenesis levels, also completely abbrogated VEGF-induced tube formation, by decreasing the number of nodes by 62% (p < 0.01), the number of junctions by 62% (p < 0.01), and the overall sprout length by 36% (p < 0.02). miR-506 treatment alone strongly, but less potently inhibited tube formation, compared to the combinatorial miR treatment, by decreasing the number of nodes by 32% (p < 0.01), the number of junctions by 32% (p < 0.01), and the overall sprout length by 11% (p < 0.05). In contrast to the above results, miR-143 did not block VEGF-induced angiogenesis, although inhibited tube formation in absence of VEGF (without reaching statistical significance).

## Discussion

CDKs are crucial for cell proliferation, with their activation regulating and/or promoting the cell cycle progression^13^. CDK1 is critical for the progression during the G2/M transition and mitosis^13,17^, and studies on CDK1 knockout mouse models revealed the irreplaceability of this gene for cell proliferation^36,37^. On the other hand, phosphorylation of retinoblastoma protein 1 (RB1) carried out by CDK4/6 is pivotal in the transition from G1 to S phase in many cancer cells^38^. Interestingly, CDK4/6-null cells were able to enter the S phase responding to serum stimulation, although at lower efficiency^39^. Thus, a combinatorial inhibition of CDK1 and CDK4/6 constitutes a promising approach to induce strong cell cycle arrest and has higher potential in inhibiting cell proliferation^37^. Among the large number of CDK inhibitors evaluated in clinical trials, only one recently succeeded to translate to patient treatment. The CDK inhibitors’ shortfalls were attributed to their semi-synthetic nature that cause significant toxicity and non-specific activity^37^.

In this study, we identified two miRs, miR-143 and miR-506 that target CDK1 and CDK4/6 respectively. miR-143 and miR-506 have previously been reported to be downregulated in lung cancer cells and constitute natural endogenous products of the cells^18,40^. Transfection of A549 cells with these two miRs efficiently downregulated the three CDKs, CDK1, 4 and 6, at the mRNA level, as detected by qPCR, and at the protein level, for CDK1 and CDK4, as detected by Western Blot (Fig. 1).

miR-143 alone preferentially downregulates the expression of CDK1, while miR-506 preferentially downregulates CDK4 and 6 (Fig. 1). This can be potentially attributed to an overall effect on the cell cycle progression by the individual miRs, causing alterations to CDK expressions that are not targeted. In contrast, the combinatorial miR treatment consistently strongly downregulated all studied genes, indicating a potential for simultaneously targeting different phases of the cell cycle (Fig. 1).

Acknowledging the ability of the combinatorial miR treatment to downregulate the expression of CDK1 and CDK4/6, we evaluated the effects of the treatment on the cell cycle progression. We utilized a standard flow cytometric analysis to identify cell distribution at the cell cycle phases^41^. Overall, we detected a significant population decrease for cells in the S phase for both the 24 and 48 h time points, following miR treatment. Although treatment of the A549 cells with the individual miRs exhibited strong alterations in the cell cycle population distribution, the combinatorial treatment consistently exhibited stronger effect, achieving statistical significance for all the studied time points. We identified a significant increase of the cell population at the G0/G1 and G2 phases, accompanied with a significant drop for the cell population at the S phase (Fig. 2). These results suggest that the combinatorial treatment induced a strong cell cycle arrest, and due to the increase of the cell population in both the G0/G1 and G2 phases, a cell cycle arrest at the G1/S and G2/M transitions could potentially take place.

The observed downregulation of the anti-apoptotic gene BCL2 (Fig. 1) prompted us to evaluate whether the combinatorial miR treatment could potentiate apoptosis. We identified a significant activation of caspase 3/7 after combinatorial miR therapy in A549 cells, an effect that was comparable to equimolar treatment of doxorubicin (Fig. 3C). Although the individual miRs alone induced caspase activation, the effect was a modest. These results are a strong indication that the combinatorial therapy initiates apoptosis. To confirm this, we performed an Annexin V/PI apoptotic analysis and identified a strong shift of the treated cell population towards the late apoptotic/necrotic stage, due to the combinatorial miR treatment. This was associated with a significant increase of the Annexin V positive cells (Fig. 3).

Subsequently, we evaluated the cytotoxic activity of the combinatorial miR treatment against A549 cells, at the same concentration as in the experiments described above, using Celltox green assay kit that permits a time-dependent evaluation of the cytotoxicity. Although the individual miRs induced a modest cytotoxic effect, the combinatorial miR treatment induced a strong cytotoxic effect (p < 0.05), greater than the addition of the cytotoxic effects of the individual miRs, potentially indicating a synergistic mechanism for the two miRs (Fluorescence intensity values average: Combo: 11636 ± 1828.5; miR-143: 3760 ± 43.5; miR-506: 5643 ± 296.5). Statistically, the observed cytotoxic effect is significantly stronger compared to the individual miRs (p < 0.05, one-tailed t-test, Fig. 3A).

Utilizing an antibody microarray technique, we analyzed the expression of proteins associated with the cell cycle, to identify the overall impact of the miRs treatment on the pathway. Although the technique is considered semi-quantitative, it is a reliable method to indicatively evaluate a pathway behavior^42^. We identified changes of the gene expressions for the specific pathway, favoring a cell cycle arrest, confirming the data presented above from the cell cycle analysis. Interestingly, the dysregulation of protein expressions, as presented in Fig. 4a and Table 2, represents an arrest exclusive to neither G1 (CDK4), nor G2 (CDK1) stages, potentially indicating that the combinatorial treatment induces cell cycle arrest in both G1 and G2 stages. Additionally, there is a difference in the observed gene expression profile for the pathway for the two time-points, 24 and 48 h. From the data presented above, the miR treatment induced the strongest downregulation of the CDK genes pre-translationally at the 24 h time point (qPCR analysis, Fig. 1a), and post-translationally at the 48 h time point (Western Blot analysis, Fig. 1b). Similarly, the microarray analysis confirms the strongest downregulation of the CDK1 and CDK4 proteins at the 48 h time point. Thus, we believe that the 48 h post-transfection results from the microarray more accurately represent the pathway behavior at the protein level due to the treatment. Nonetheless, for the two time points, there is an upregulation of proteins that negatively regulate the cell cycle progression and downregulation of proteins that promote cell cycle progression. These results confirm that the proposed treatment has significant capacity in halting the cell cycle.

Subsequently, we performed a more elaborate analysis of the mechanistic behavior of the cells due to the combinatorial miR treatment, through RNA-sequencing and bioinformatic analysis on the data obtained. The RNA-sequencing confirmed the CDK1, CDK4, CDK6 and BCL-2 downregulation due to the combinatorial miR treatment (vs. untreated cells), similarly to the qPCR analysis. Furthermore, the cell cycle pathway heatmap was strongly dysregulated, with the majority of the cell cycle-associated genes being downregulated (Fig. 4b, Supplementary Fig. 5). We performed mechanistic analysis of the observed gene dysregulations using the IPA software. The software predicted a cell cycle arrest in both G1/S and G2/M transition phases, with the overall gene dysregulation indicating an abnormal cell cycle and cell death (Fig. 4c). Through further analysis with the IPA software, we identified the 10 pathways with the lowest activation z-score and 10 pathways with the highest activation score (Fig. 5a,b).

Interestingly, the pathways that had the lowest activation score are directly related to cell apoptosis and abnormal cell cycle progression (Fig. 5a,b). Indicatively, the integrin signaling pathway demonstrated the lowest activation score. This pathway is associated with the cell cycle progression, with integrins constituting a spatial checkpoint for transitioning from G1 to S phase^43^. Furthermore, the integrin signaling regulates the Erk/Mek pathway, which in turn contributes to cyclin D1 expression and the transition through G1/S phases, with pathway activation promoting cell cycle progression overall^43^. Not surprisingly, IPA predicted a strong negative z-score for the Erk/MAPK signaling. It is important to add that the integrin and Erk signaling are important for the downregulation of the p21-family CDK inhibitors (i.e., p21^CIP1^, also known as CDKN1A)^44^. In our data, IPA predicted strong negative z-scores for the integrin and Erk pathways (indicating a reduced pathway activity), while the RNA-sequencing data exhibit a 2.1-fold increased expression of CDKN1A. In contrast, the relative expression of the integrins according to the RNA-sequencing data (ITGA11, ITGA2, ITGA3, ITGA5, ITGAE) was not impacted, with the majority of the integrin genes being excluded from the analysis according to the set cutoffs. The only exceptions were ITGB1, which was downregulated by 89%, and ITGAX having a 3.59-fold upregulation.

In contrast, the pathway with the highest activation score was the PTEN signaling, according to IPA. PTEN is recognized as a negative regulator for the PI3-kinase/Akt signaling pathway, which controls the cell cycle progression and cell death, while previously has been shown that Pten-/- cells demonstrate an increased growth rate and advanced entry into S phase^45^.

The described pathways impact the cell cycle progression and cell survival. Not surprisingly, the IPA software predicted that the induced gene dysregulation would negatively impact the cell movement, cell survival, and promote cell senescence, apoptosis and cell death (Fig. 4c). It is important to note that the BRCA1 pathway demonstrated a large number of downregulated genes, according to IPA. BRCA1 loss of function is associated with diminished capacity for DNA damage-induced cell cycle checkpoint activation (primarily in breast cancers)^46,47^. Although the BRCA1 function is important and complete loss of function can be detrimental, downregulation of the wild-type BRCA1 in cancer cell populations can provide benefits in the therapeutic utility of PARP-inhibitor monotherapy. In fact, combination of inhibition of CDK1 and PARP in BRCA-proficient cells resulted in reduced colony formation, tumor xenograft growth inhibition and tumor regression with prolonged survival in a mouse lung adenocarcinoma model, despite BRCA1 downregulation^48^. According to our results, a combination of BRCA1, CDK1 and PARP1/2 downregulation takes place, which supports the observed behavior of the A549 cells presented above by the IPA software. Nonetheless, further evaluation is required.

Microarray analysis indicated a downregulation of CDK5 due to miR-143/-506 treatment. CDK5 regulates endothelial migration and angiogenesis, recognized as a potential target for anti-angiogenic therapy^32^. Additionally, the IPA software indicated an impairment of cell mobility, invasion and angiogenesis (Fig. 5c, Supplementary Fig. 7), as well as predicted the Vascular Endothelial Growth Factor (VEGF) gene as an upstream transcriptional regulator, with z <−2 (Fig. 5d). VEGF is a known angiogenic growth factor and frequently consists the main target of anti-angiogenic therapies^33,34^. These results prompted us to evaluate the potential anti-angiogenic effect of the treatment using a standard tube formation assay.

The combinatorial miR treatment blocked tube formation *in vitro* in the absence of VEGF, as assessed by the decrease in the number of nodes, number of junctions and overall sprout length (Fig. 6), in an *in vitro* angiogenesis assay^35^. Individually, miR-506 demonstrated slightly less potent effect than the combinatorial treatment, whereas miR-143 demonstrated a modest effect. In contrast, the combinatorial miR treatment completely abbrogated VEGF-induced tube formation. The effect of miR-506 was slightly less potent, whereas miR-143 was less potent on inhibiting basal or VEGF-induced angiogenesis levels.

There is contradicting information in the literature regarding the role of miR-143′s on angiogenesis, with some researchers indicating an increase in angiogenic activity^49^, while others indicating an anti-angiogenic effect of miR-143^50^, which undoubtedly has resulted to an interesting debate^51^. From our preliminary work, we identify that miR-143 has anti-angiogenic activity (conditionally), although it is modest. Potentially, a higher concentration of miR-143 is required to achieve significant effect, and an alternative mechanism of action may take place, compared to miR-506 that reduces VEGF-induced tube formation. The combinatorial miR treatment had a stronger effect than the individual miRs alone in inhibiting tube formation. Furthermore, the combinatorial treatment completely abrogated VEGF’s effect, that the individual miRs were unable to perform. Further analysis is required to fully identify the molecular pathways affected in the primary endothelial cells by the combinatorial treatment. In summary, the combinatorial treatment was collectively stronger in inhibiting tube formation and potentially angiogenesis, which adds an additional therapeutic benefit for cancer treatment to the proposed treatment.

## Conclusion

In this study, we evaluated the combinatorial therapy of miR-143 and miR-506 against lung cancer cells. The combinatorial treatment targets the cell cycle progression and downregulates CDK1, CDK4 and CDK6 expression, leading to cell cycle arrest, as well as induces strong cytotoxic and apoptotic activity in A549 NSCL cells. The treatment induced a strong inhibition of tube formation *in vitro*, indicating an associated anti-angiogenic potential. This multi-faceted and strong activity of the proposed miR treatment is promising, and further studies will improve our understanding on the potential of this approach as an anticancer therapy for lung cancer.

## Materials and Methods

### Materials

Cell culture reagents were purchased from Gibco^TM^ (Life technologies, Carlsbad, CA). ECGS was purchased from BD Biosciences (Cat#356006, San Jose, CA), heparin solution was purchased from Hospira (Lake Forest, IL). MicroRNAs were purchased from ABM (Richmond, BC, Canada). Scramble-siRNA was purchased from Ambion (Foster, CA). Opti-MEM, Lipofectamine 2000 reagent and VEGF were purchased from ThermoFisher. Quick-RNA miniprep kit was purchased from Zymo Research (Irvine, CA). Reduced Growth Factor (RGF)-Basement Membrane Extract was purchased from Trevigen (Gaithersburg, MD). Bovine Serum Albumin (BSA), Tris-HCl, NaCl and Nonidet P-40, phenylmethylsulfonyl fluoride, aprotinin, leupeptin and other chemicals were purchased from Fisher or Sigma. Biorad-DC protein assay kit was purchased from Bio-Rad (Hercules, CA). Anti-CDK1, -CDK4, -β-tubulin and secondary antibodies were purchased from Abcam (Cambridge, MA).

### Cell culture and transfection

NSCLC A549 cell line was cultured in DMEM/F12K medium, supplemented with 10% fetal bovine serum and 1% penicillin/streptomycin. Cells were maintained at 37 °C with 5% CO_2_ in a humified environment, following standard protocols. Human Umbilical Vein Endothelial Cells (HUVECs) were isolated from human umbilical cords in accordance with the relevant guidelines and regulations from the Texas Tech University Health Sciences Center Institutional Review Board (IRB#A15-3891). The protocol for HUVEC isolation was approved by the TTUHSC IRB committee, and informed consent was obtained from all subjects. HUVECS were used at passages 1-6. HUVECs were grown as a monolayer in medium M199 that was supplemented with 15% Fetal Bovine Serum (FBS), 150 µg/ml Endothelial Cell Growth Supplement (ECGS), 5 U/ml heparin sodium and 1X Antibiotic-Antimycotic solution^52^.

Cells were transfected with miR-143 (-3p) and miR-506 (-3p) mimics and scramble, using Lipofectamine 2000, following manufacturer’s instructions. Briefly, we cultured the cells in T25 cm^2^ flask/6well/96well plate, depending to the experimental design, and transfected with the respective miR mimics or scramble at the concentration of 100 µM using Lipofectamine 2000, suspended in optimem reduced serum and incomplete media. Following 6 h of incubation, the media containing nucleic acids was replaced with complete media and cells were further incubated. We harvested the transfect cells 24 or 48 h post transfection. Untreated cells were cultured under same conditions.

### Quantitative RT-PCR (qPCR) analysis

Total RNA was isolated using Quick-RNA miniprep kit, following the manufacturer’s instructions, and RNA concentration was determined using Nanodrop. cDNA was synthesized using Verso cDNA Synthesis Kit, following manufacturer’s protocol. Quantitative real-time PCR was performed using PowerUP SYBR Green Master Mix (Applied Biosystems, Carlsbad, CA) on a Bio-Rad CFX96 Real time PCR system (Bio-Rad systems, USA). We utilized primers designed specifically for the detection of CDK1, CDK4, CDK6, BCL-2, and GAPDH (S.I. Table 1), with the latter serving as the reference gene. All results were normalized to untreated cells and differential gene expression was calculated. Scramble siRNA with lipofectamine were used for comparison. All p-values are in comparison to untreated (control) samples. Data analysis was performed using the −ΔΔCt method.

### Protein expression using Western Blot analysis

We extracted protein content from A549 cells transfected with miR-143 and/or miR-506 at the 24 and 48 h time point post transfection, and performed western blot analysis, following previously established protocols^41^. Anti-CDK1 and anti-CDK4 antibodies were used to identify the respective proteins. Anti-beta Tubulin antibody was used as a loading control. After blotting, protein bands were identified using chemiluminescent substrate (Pierce ECL western blotting substrate, Rockford. IL) and visualized under a Chemidoc imaging system (Bio-Rad system, USA).

### Cell cycle assay

The analysis of the cell cycle was performed with a flow cytometric technique, using previously established protocols^41^. Briefly, we harvested A549 cells transfected with miR-143 and/or miR-506 at 24 and 48 h post transfection, and fixed them with 70% of ethanol. Subsequently, cells were stained with propidium iodide (MP Biomedials, LLC, Illkirch, France) and analyzed using a BD FACSCalibur Flow Cytometer, along with Cellquest Pro software (BD Biosciences, Franklin Lakes, USA). The data were further analyzed using ModFit LT 5.0. The cell cycle data are presented as the percentage of cell distributions in the different phases (G0/G1, S and G2).

### Apoptosis Assay

We transfected A549 cells with miR-143 and/or miR-506, as described above, and harvested the cells 24 and 48 h post transfection. Subsequently, we stained the cells with FITC-Annexin V and Propidium iodide (PI) and analyzed them using a BD FACSCalibur Flow Cytometer to determine apoptotic behavior due to treatment, using previously established protocols^41^. Untreated cells, and cells treated with scramble + lipofectamine and lipofectamine only were used as negative controls.

### Detection of Caspase 3/7 activity

We utilized the Apo-One Homogeneous Caspase 3/7 assay kit (Promega) to identify levels of Caspase 3/7 in A549 cells transfected with miR-143 and/or miR-506 at the 24 and 48 h time points post transfection, according to the manufacturer’s protocol.

### Antibody cell cycle microarray analysis

We utilized a semi-quantitative antibody microarray, obtained from Full Moon Biosystems (Sunnyvale, CA) to detect the relative expression of several proteins. The microarray slide was specifically designed to detect 60 cell cycle associated proteins (Prod. #: ACC058), including reference genes, with 6 replicates for each antibody. We transfected A549 cells with miR-143 and miR-506 and 24 and 48 h post transfection, and proteins samples were extracted. Biotinylation and hybridization of protein samples were carried out according to manufacturer’s instructions. Fluorescent-labeled Cy-3-streptavidin dye (GE Healthcare- UK) was used to detect protein levels. We scanned the microarray using the Pro ScanArray HT Microarray Scanner (PerkinElmer, MA), and analyzed the data using the ScanArray Express software.

### Cytotoxicity assay

We assessed the miR-induced cell death on A549 cells, using the Celltox green assay kit (Promega), according to the manufacturer’s instructions. Briefly, we seeded A549 cells to a 96-well plate and transfected them with miR-143 and/or miR-506, as described above. In addition, CellTox green dye (Cyanine dye) was included to the mix, which binds to DNA of dead cells and fluoresces proportionately to the number of dead cells (10 µl of CellTox dye per 5 ml of cell culture media). After 6 h of transfection, the media was supplemented with complete culture media in each well. 8 µl/well of lysis solution (supplied by vendor) was used as positive control. Fluorescence was measured by excitation at 485 nm and detection at 528 nm, using a Biotek synergy H1 plate reader (Winooski, VT) at different time points to detect cell death as a function of time.

### RNA-sequencing for gene expression analysis

RNA-sequencing took place in collaboration with the University of Texas Southwestern Medical Center, McDermott Center Next Generation Sequencing Core. We transfected A549 cells with miR-143 and miR-506 using lipofectamine, as described above, and harvested the cells 24 h post transfection. We extracted the RNA using Quick-RNA MiniPrep kit. To determine RNA level of degradation, the samples were run on an Agilent Tapestation 4200, and only high quality RNA was used (RIN Score 8 or higher, S.I. Figs 3 and 4). RNA concentration was determined using a Qubit fluorimeter. 4 µg of DNAse treated RNA samples were then prepared using the TruSeq Stranded mRNA Library Prep Kit from Illumina (San Diego, CA). Prior to strand specific cDNA synthesis, poly-A RNA is purified and fragmented, and cDNA was then a-tailed and indexed adapters were ligated. Samples were subsequently amplified by PCR and purified using AmpureXP beads. Successful cDNA synthesis was validated on the Agilent Tapestation 4200. Samples were quantified by Qubit then run on a Illumina NextSeq. 500 using V2 reagents. Fastqc (v0.11.2)^53^ and fastq_screen (v0.4.4)^54^ were used to check Fastq files quality, and quality trimming took place using fastq-mcf (ea-utils/v1.1.2-806)^55^. Trimmed fastq filers were mapped and duplicates were marked. Differential expression analysis on the read counts was performed using EdgeR^56^, between treated and control samples. Samples for RNA-sequencing were analyzed in duplicates.

### Pathway analysis from RNA-sequencing

The RNA-sequencing analyzed the expression of 18,533 genes. Pathways were selected from KEGG PATTHWAY database (http://www.genome.jp/kegg/pathway.html) that were associated with lung cancer. All relative gene expression values were distributed to their respective pathways, and heatmaps were produced (Supplementary Fig. 5). The expression patterns of specific pathway genes were clustered with Gene Cluster 3.0, so that similarly-regulated genes were grouped together, and then displayed in a heatmap with Java Tree view 1.1.6r4^57^.

Ingenuity Pathway Analysis (IPA, Qiagen) was performed using all genes with a counts-per-million (CPM) value >1 at least at one of the control duplicates, False Discover Rate (FRD) value < 0.05, and log2-fold-change (logFC) values that were less than -0.4 and larger than 0.6. The remaining genes subsequent to the above cut-odds had p-values < 0.02. The final number of genes analyzed by the IPA software was 2,803. The RNA-sequencing files were uploaded to the Genbank,(SUB: SUB3721227) with accession number SRP133420.

### Matrigel tube formation assay

Matrigel tube formation assay was performed with transfected HUVECs 36 h post-transfection. Briefly, wells of a 96-well culture plate were coated with 0.04 ml RGF Basement Membrane Extract (Trevigen, Cat#3433) and were left to polymerize for 20 min at 37 °C. Then, 1 × 10^4^ cells suspended in 100 µl of M199 were added to the respective wells. For each treatment, 2 ng/ml of VEGF (Cat#PHC9394, ThermoFisher) was used as positive control. Following 6 h of incubation at 37 °C, pictures of the wells were captured using a bright field microscope connected with a digital camera at 4X magnification^58^ and later analyzed for number of nodes, number of junctions and total sprout length using the ImageJ software and “Angiogenesis analyzer” plug-in^59^.

### Statistical Analysis

Statistical analysis was performed using Microsoft Excel t-test to determine any significant difference between treatment groups. The two-tailed unpaired t-test (unless stated otherwise) was used to compare the mean values ± standard errors; p values < 0.05 were considered statistically significant.

### Data availability

All data generated or analyzed during this study are included in this published article (and its Supplementary Information files).

## Electronic supplementary material

Supplementary Information
