# Supplementary material for: Multipronged activity of combinatorial miR-143 and miR-506 inhibits Lung Cancer cell cycle progression and angiogenesis in vitro
Source: Sci Rep. 2018 Jul 12;8:10495. doi: 10.1038/s41598-018-28872-2 (PMC6043488; doi:10.1038/s41598-018-28872-2)
Supplement: Supplementary file 1 — Supplementary Information [file 41598_2018_28872_MOESM1_ESM.pdf]

Multipronged activity of combinatorial miR-143 and miR-506 inhibits Lung Cancer cell cycle progression and angiogenesis *in vitro*

A K M Nawshad Hossian[1], Md. Sanaullah Sajib[2], Paul E. Tullar[3], Constantinos M. Mikelis[2], George Mattheolabakis[1*]

[1]Department of Basic Pharmaceutical Sciences, School of Pharmacy, University of Louisiana at Monroe

[2]Department of Biomedical Sciences, School of Pharmacy, Texas Tech Health Sciences Center

[3]Department of Obstetrics and Gynecology, School of Medicine, Texas Tech University Health Sciences

Supplementary Table 1: Cell cycle distribution for cells treated with miR-143 and/or -506 at the 24 and 48 h time points post transfection

	**G0/G1**	**S**	**G2/M**
**24h**			
**Untreated**	47	41	11
**miR-143**	63	22	14
**miR-506**	63	23	14
**miR-143+506**	71	17	13
**48h**			
**miR-143**	66	21	13
**miR-506**	76	13	11
**miR-143+506**	75	12	13

Supplementary Table 2: Primer sequences for identifying different gene expressions by qRT-PCR

Target Gene	Primer direction	Primer sequence
CDK1	Forward	TTTTCAGAGCTTTGGGCACT
CDK1	Reverse	CCATTTTGCCAGAAATTCGT
CDK4	Forward	TATGAACCCGTGGCTGAAAT
CDK4	Reverse	CATCAGCCGTACAACATTGG
CDK6	Forward	CGTGGTCAGGTTGTTTGATGTG
CDK6	Reverse	ACTCGGTGTGAATGAAGAAAGTCC
GAPDH	Forward	TGCACCACCAACTGCTTAGC
GAPDH	Reverse	GGCATGGACTGTGGTCATGAG
BCL2	Forward	CCTTGTGGATGACTGAGTACC
BCL2	Reverse	AGCCAGGAGAAATCAAACAGAG

Supplementary Figure 1: Cell cycle distribution for cells treated with scramble siRNA+lipofectamine compared to control

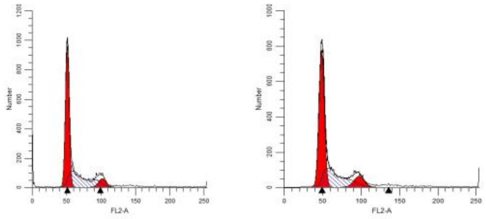

Control 24h

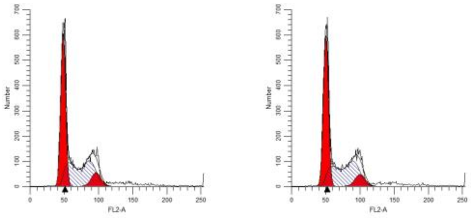

Control 48h

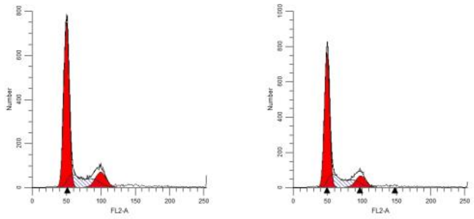

Scramble 24h

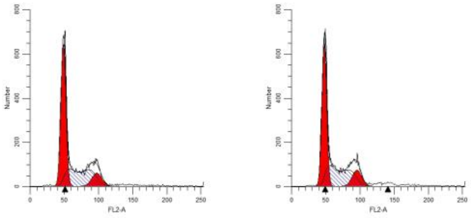

Scramble 48h

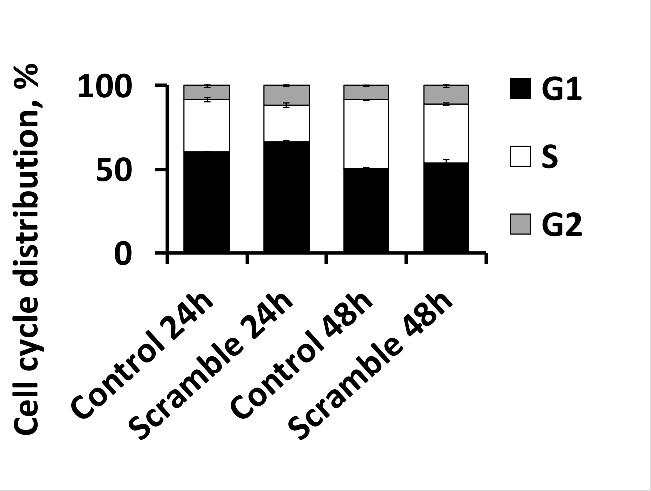

Sample	G0/G1	S	G2/M
Control 24h	60.27	32.54	7.19
Control 24h	60.48	30.01	9.51
Scramble 24h	67.12	20.7	12.17
Scramble 24h	65.38	23.66	10.96
Control 48h	49.81	41.33	8.86
Control 48h	51.3	40.62	8.08
Scramble 48h	55.65	34.2	10.16
Scramble 48h	52.24	35.95	11.81

Supplemental Figure 2: Annexin V/PI apoptosis assay for cells treated with scramble siRNA+lipofectamine and lipofectamine alone compared to control

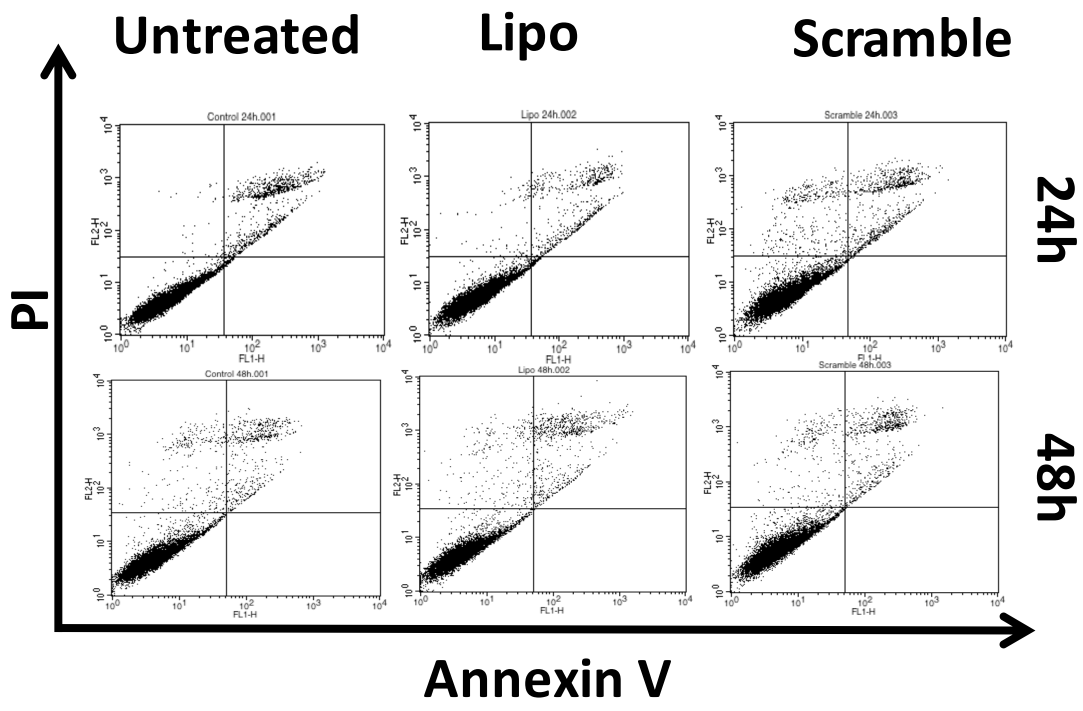

Supplementary Figure 3: RNA quality assessment prior RNA sequence (first duplicate for untreated and miR treated samples)

First Replicates

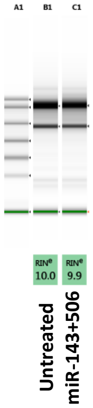

Untreated

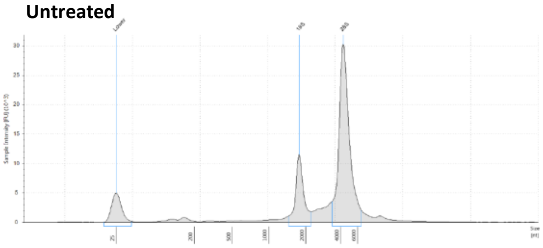

Sample Table

Well	RINe	28S/18S (Area)	Conc. [ng/µl]	Sample Description	Alert	Observations
B1	10.0	3.5	221	NH-1		

Peak Table

Size [nt]	Calibrated Conc. [ng/µl]	Assigned Conc. [ng/µl]	Peak Molarity [nmol/l]	% Integrated Area	Peak Comment	Observations
25	40.0	40.0	4710	-		Lower Marker
1778	38.0	-	62.9	22.15		18S
4279	134	-	91.9	77.85		28S

miR-143+506

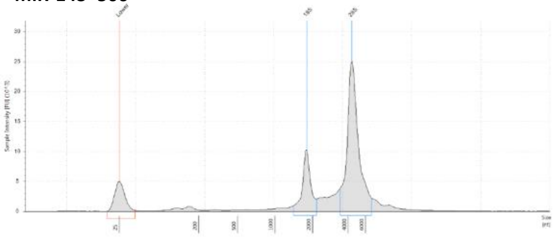

Sample Table

Well	RINe	28S/18S (Area)	Conc. [ng/µl]	Sample Description	Alert	Observations
C1	9.9	3.4	203	NH-2		

Peak Table

Size [nt]	Calibrated Conc. [ng/µl]	Assigned Conc. [ng/µl]	Peak Molarity [nmol/l]	% Integrated Area	Peak Comment	Observations
25	40.0	40.0	4710	-		Lower Marker
1793	34.2	-	56.2	22.74		18S
4347	116	-	78.7	77.26		28S

Supplemental Figure 4: RNA quality assessment prior RNA sequence (second duplicate for untreated and miR treated samples)

Second Replicates

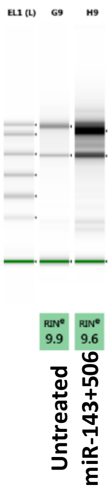

Untreated

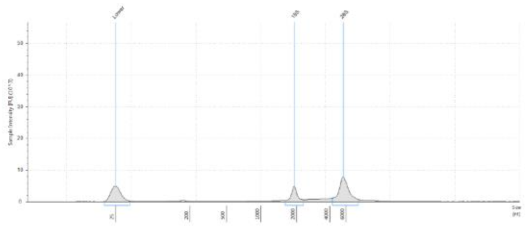

Sample Table

Well	RINe	28S/18S (Area)	Conc. [ng/µl]	Sample Description	Alert	Observations
G9	9.9	2.6	64.6	NH-1_2 1:10		

Peak Table

Size [nt]	Calibrated Conc. [ng/µl]	Assigned Conc. [ng/µl]	Peak Molarity [nmol/l]	% Integrated Area	Peak Comment	Observations
25	40.0	40.0	4710	-		Lower Marker
1923	12.4	-	19.0	27.96		18S
5616	31.9	-	16.7	72.04		28S

miR-143+506

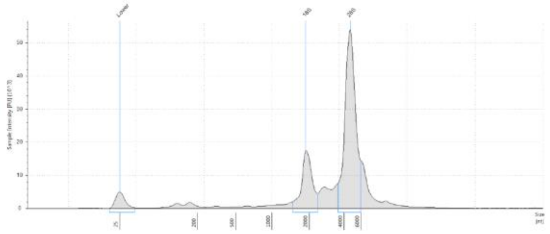

Sample Table

Well	RINe	28S/18S (Area)	Conc. [ng/µl]	Sample Description	Alert	Observations
H9	9.6	3.1	490	2_20		

Peak Table

Size [nt]	Calibrated Conc. [ng/µl]	Assigned Conc. [ng/µl]	Peak Molarity [nmol/l]	% Integrated Area	Peak Comment	Observations
25	40.0	40.0	4710	-		Lower Marker
1883	81.2	-	127	24.11		18S
4636	256	-	162	75.89		28S

Supplementary Information Figure 5: Representative heatmaps of the pathways associated with lung cancer

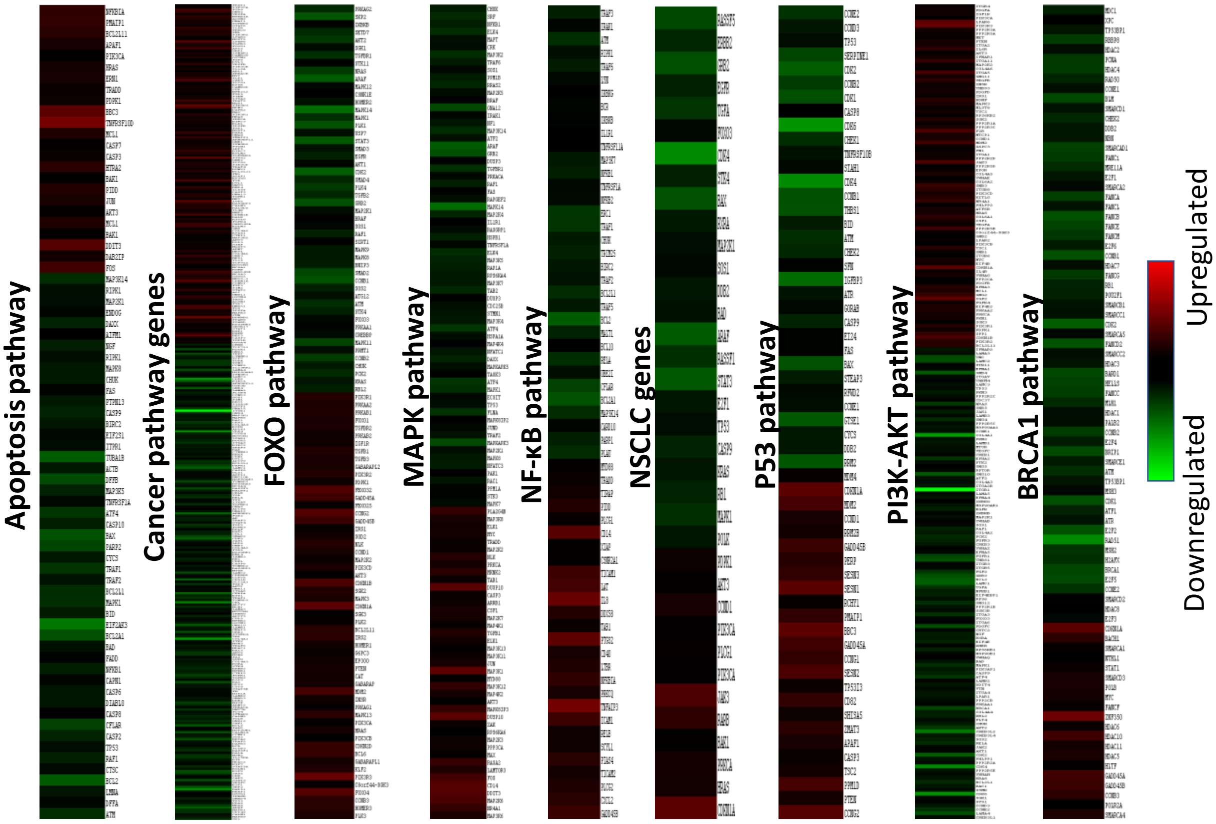

Supplementary Figure 6: Cell cycle pathway and predicted functions, as presented by IPA

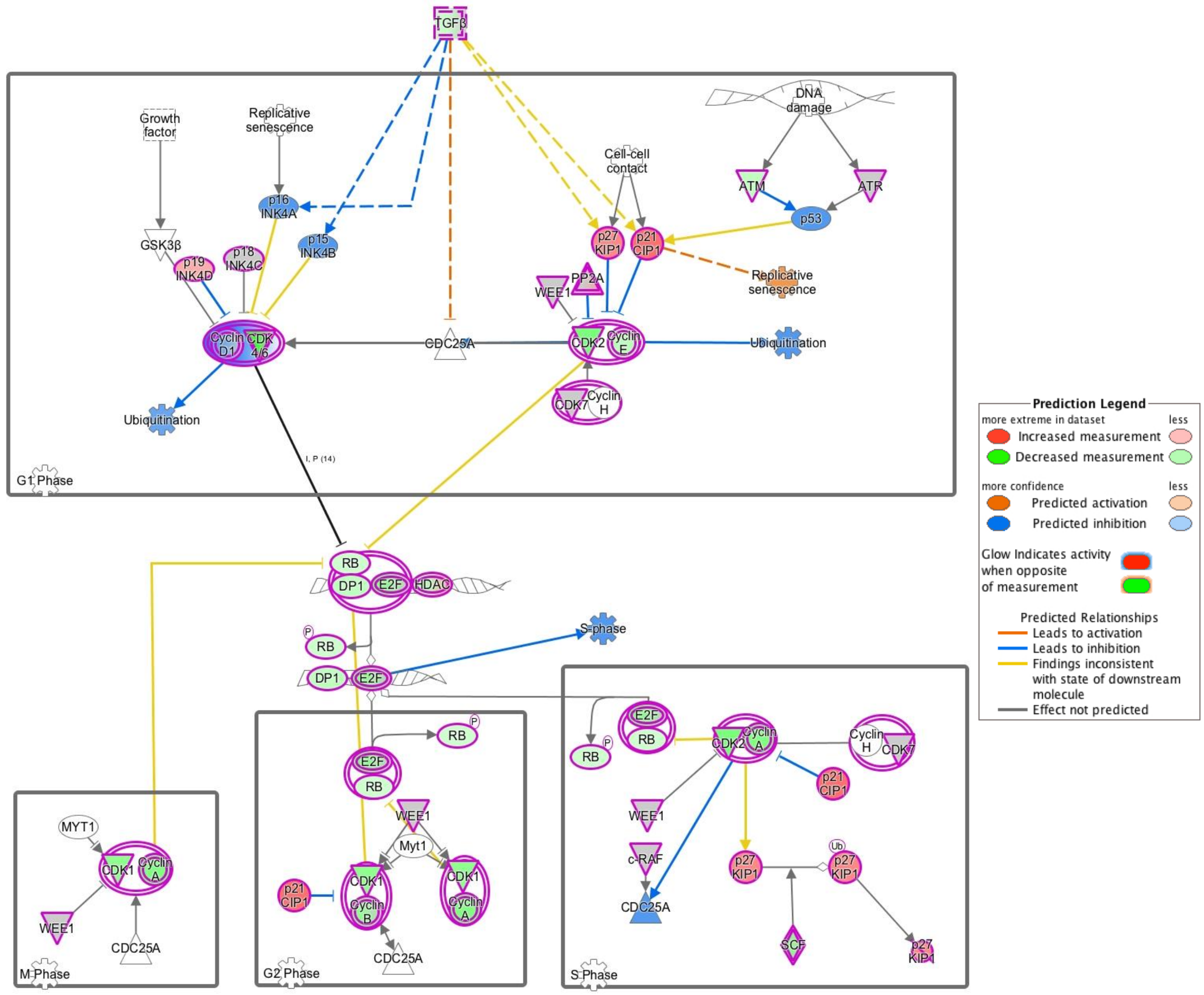

Supplementary Figure 7: Predicted functions due to miR-143 and miR-506 treatment, as predicted by IPA software

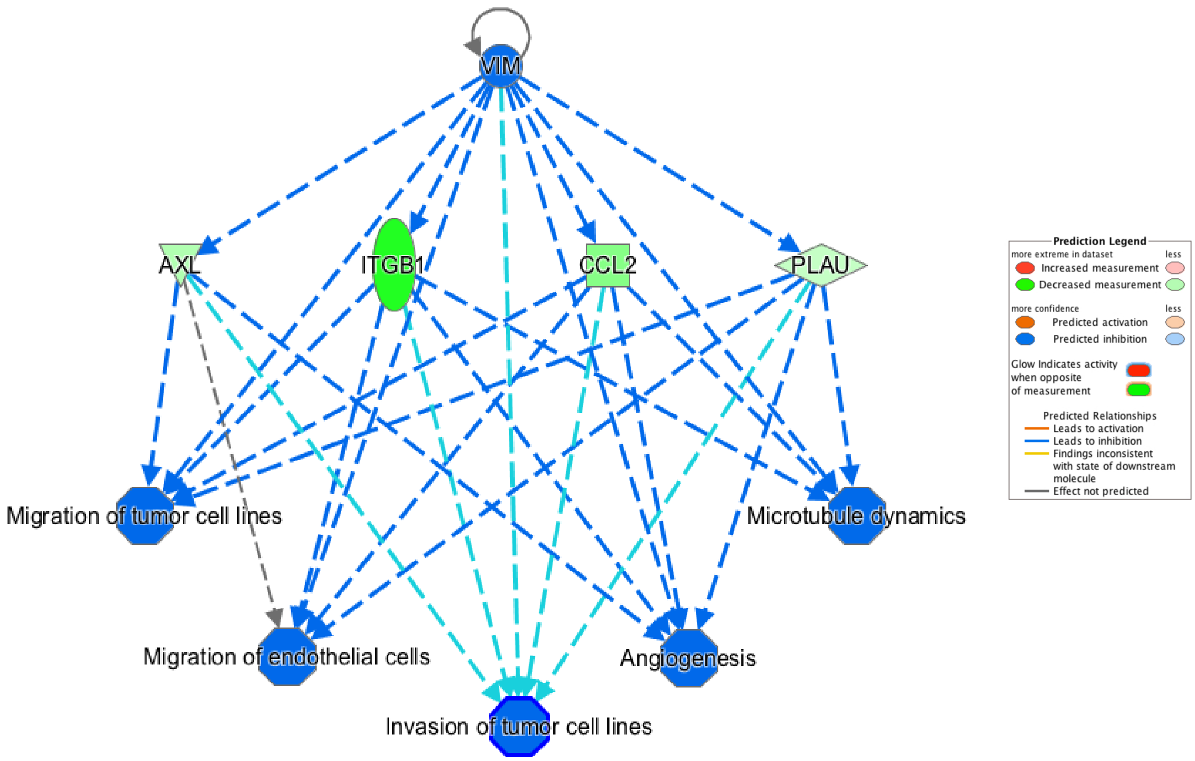

Supplementary Figure 8: BRCA1 pathway and predicted functions, as presented by IPA software, due to miR-143 and miR-506 treatment

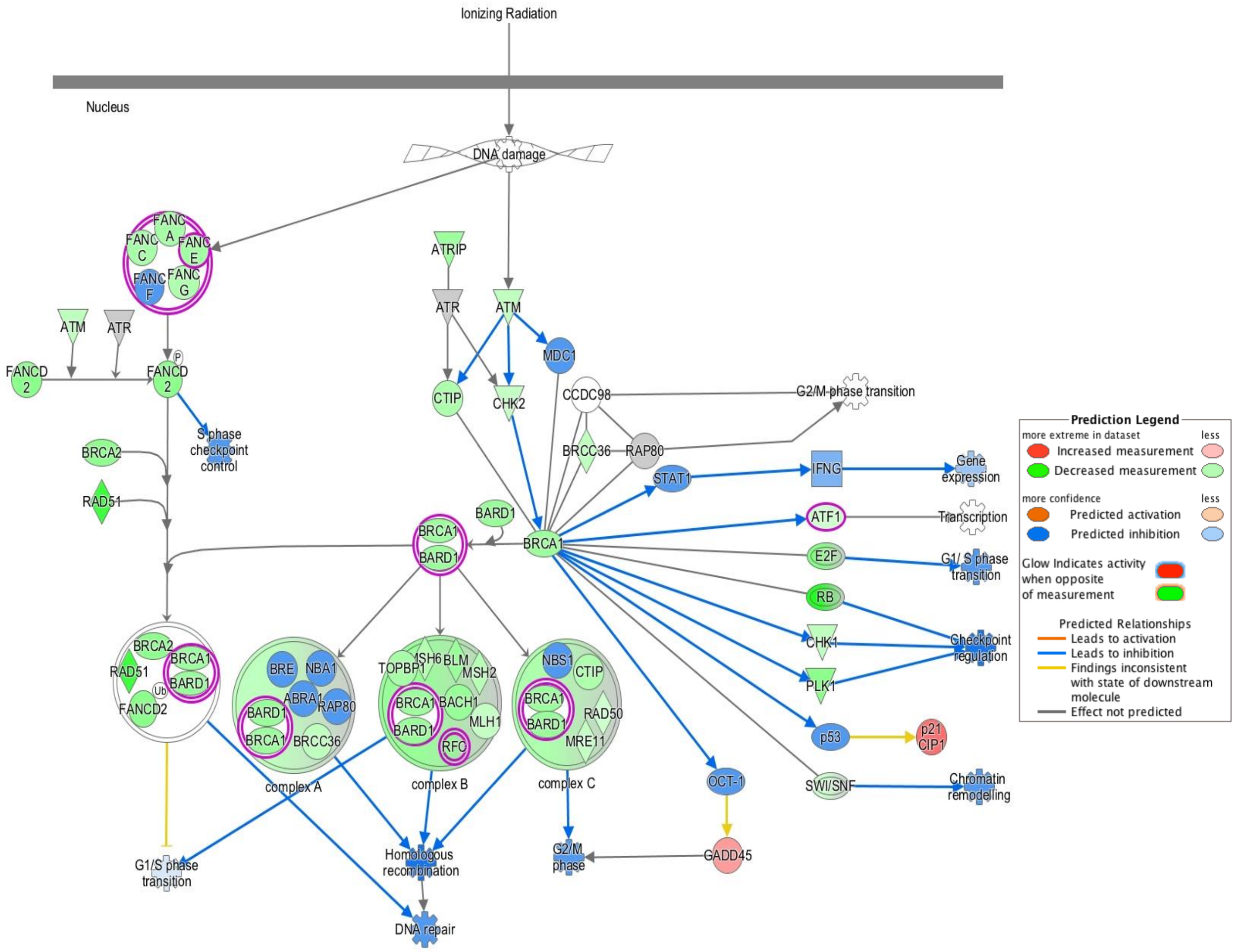

Supplementary Figure 9: Predicted apoptosis function in A549 cells due to the miR-143 and miR-506 treatment, as presented by IPA. (z-score = 3.6)

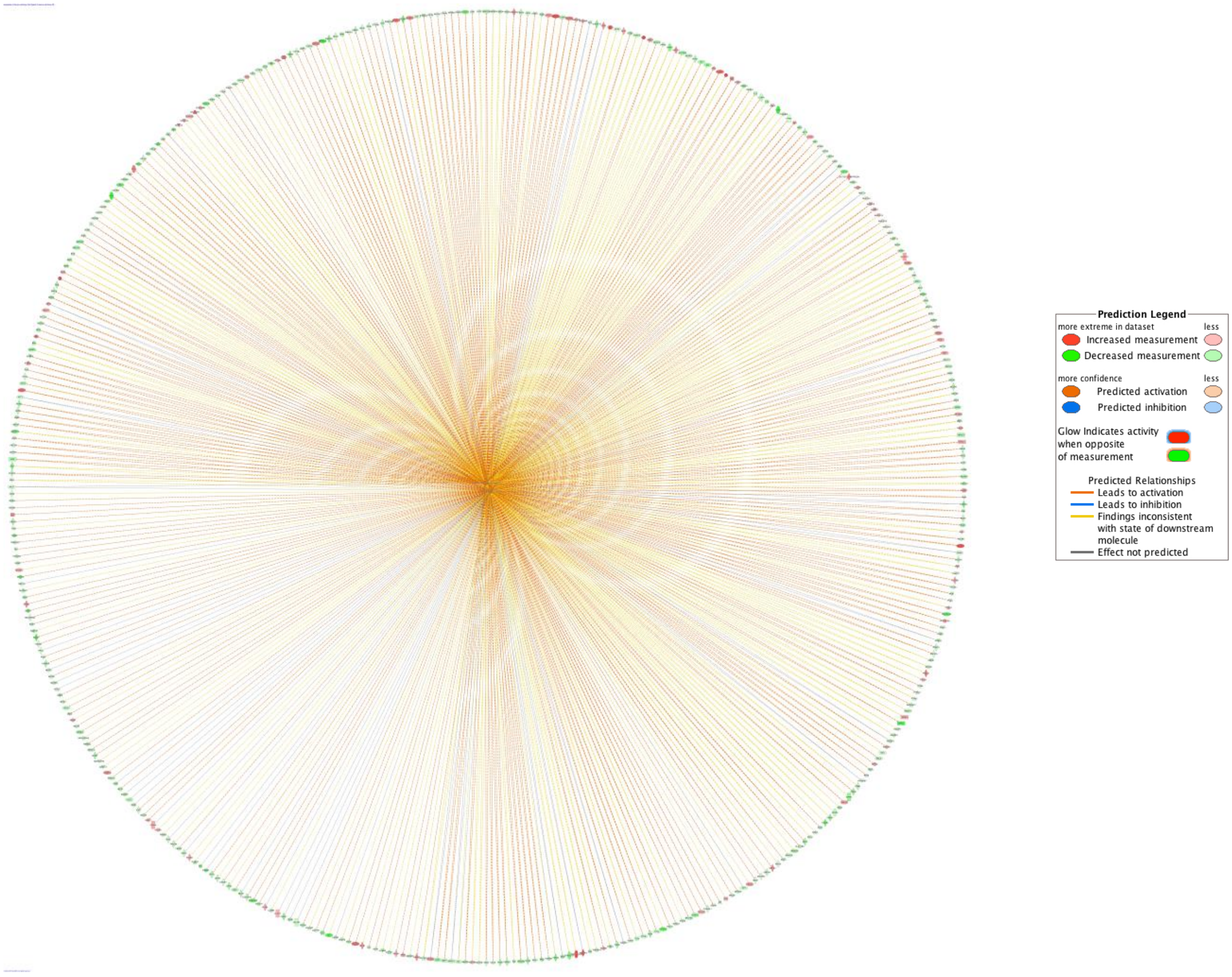

Supplementary Figure 10: *(left)* Predicted S phase of tumor cell lines (z-score = -3.118); *(right)* Predicted M phase (z-score = -2.069), as provided by IPA

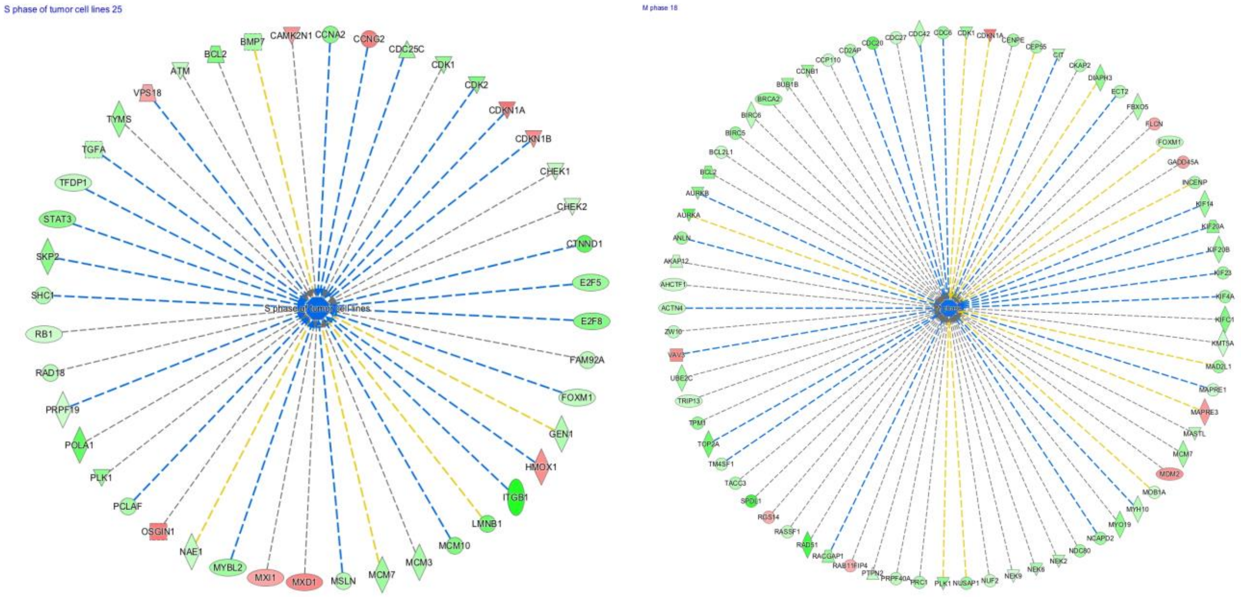

Supplementary Figure 11: Full images from Western blot analysis

CDK1	b-tubulin for CDK1 blot
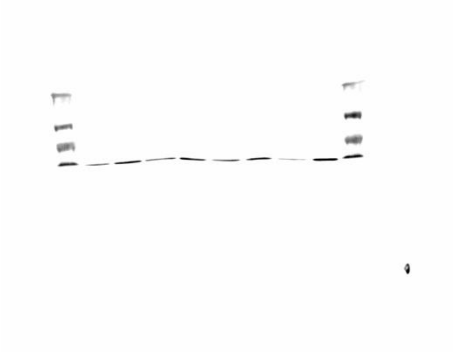	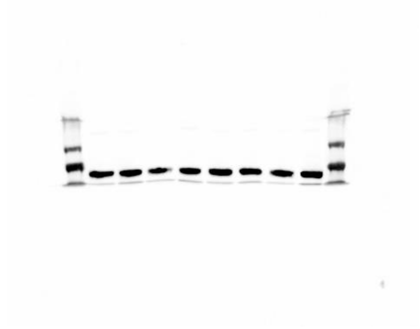
CDK4	b-tubulin for CDK4 blot
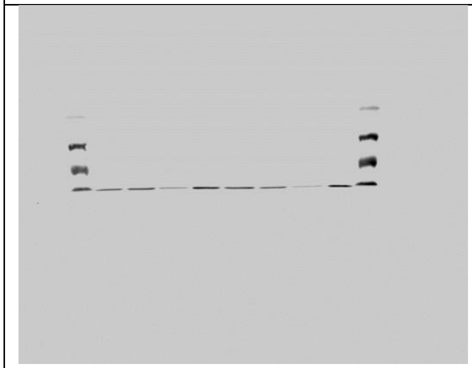	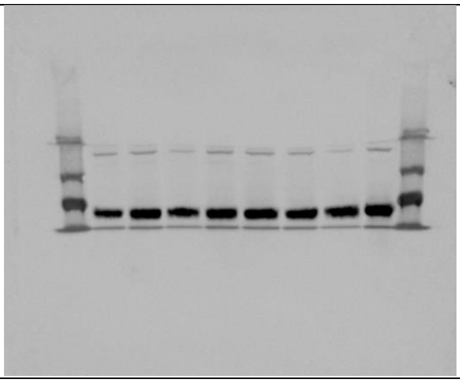
